# Endoscopic Submucosal Dissection of Seborrheic Keratosis-Like Lesion of the Esophagus: A New Entity?*

**DOI:** 10.5146/tjpath.2018.01447

**Published:** 2020-01-15

**Authors:** Nese Ekinci, Eylül Gün, Fatih Aslan

**Affiliations:** Department of Pathology, Izmir Katip Celebi University Ataturk Training and Research Hospital, Izmir, Turkey; Department of Gastroenterology, Koc University Hospital, Istanbul, Turkey

**Keywords:** Endoscopic submucosal dissection, Esophagus, Seborrheic keratosis

## Abstract

Seborrheic keratosis, one of the most common lesions of the epidermis, is rarely seen on mucosal surfaces. We report a case of a distinctive epithelial neoplasm of the esophagus showing close resemblance to seborrheic keratosis that was resected with endoscopic submucosal dissection. A 65-year-old patient’s previous esophageal biopsy showed suspicious low grade dysplasia and the patient was referred for endoscopic submucosal dissection of a flat lesion in the mid-esophagus. Macroscopic examination revealed a well circumscribed, pigmented and elevated lesion with a diameter of 20 mm. Microscopically, the lesion was well circumscribed, with plaque-like elevation, and showed hyperkeratosis, acanthosis, and papillomatosis. Broad coalescing solid sheets and interconnecting trabeculae of basaloid cells were the consistent feature throughout the lesion. Squamous eddies and occasional central keratinization were present. Mitotic activity and koilocytes were not identified. Immunohistochemically, the lesion showed diffuse nuclear positivity with p63 and negativity with p16. Ki-67 index was confined to the basal cell layer. With the help of histopathologic and immunohistochemical findings, we diagnosed this morphologically benign case as “seborrheic keratosis-like lesion of the esophagus”. It should be kept in mind that seborrheic keratosis-like lesions might be rarely seen on mucosal surfaces such as the esophagus. Endoscopic submucosal dissection is a new, curative, and safe endoscopic resection technique in en-bloc resection of superficial esophageal lesions. To our knowledge, this is the first case of the aforementioned lesion in the esophagus being resected with endoscopic submucosal dissection.

## INTRODUCTION

Seborrheic keratosis is one of the most common lesions in dermatopathology but it is rarely seen on mucosal surfaces (1). Curative endoscopic resection methods such as endoscopic mucosal resection (EMR) and endoscopic submucosal dissection (ESD) are new resection techniques initially developed in Japan and allow *en bloc *resection and treatment of superficial gastrointestinal lesions. Although application of ESD to the esophagus is limited in early stage esophageal neoplasia because of its greater technical difficulty, it avoids the high morbidity and mortality rates of surgical treatment (2) and offers a highly effective, safe and less expensive way for the detection and treatment of esophageal neoplastic lesions. It is considered the cornerstone of endoscopic treatment of Barrett’s esophagus and early squamous cell carcinoma of the esophagus (3). It is therefore increasingly and successfully in use all around the world. We report a case of distinctive epithelial neoplasm of the esophagus, which shows close resemblance to seborrheic keratosis, one of the most common benign epidermal tumors, resected with ESD.

## CASE REPORT

**Figure 1:**
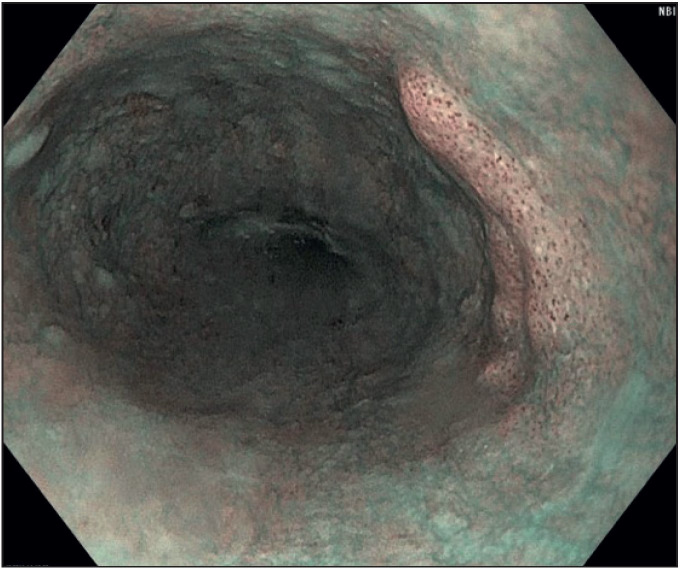
Endoscopic view of the flat lesion with a diameter of 20 mm at approximately 28 cm from the incisor teeth of the esophagus.

**Figure 2:**
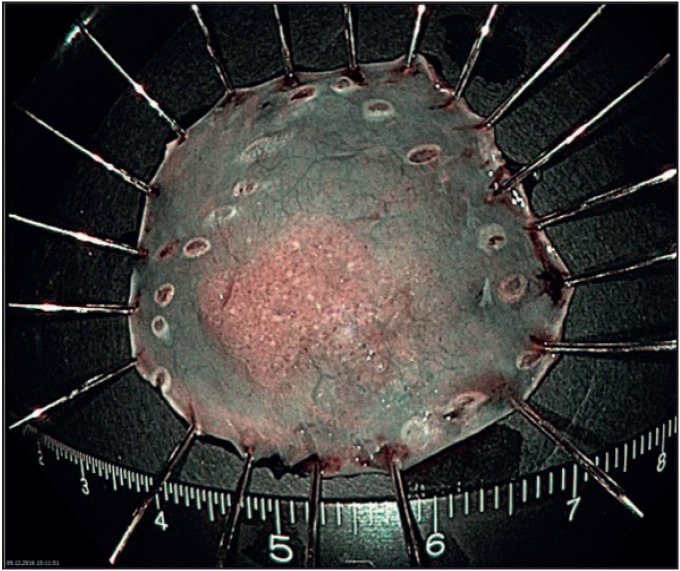
Pinned endoscopic submucosal dissection specimen.

A 65-year-old man with slight dyspnea was referred to the gastroenterology clinic of a tertiary hospital in 2013. Gastro-esophagoscopy was done and no abnormality was found. The follow-up endoscopic biopsy in November 2016 showed low grade dysplasia and the patient was referred to our hospital for further examination and treatment. After pre-procedural assessment with narrow band imaging and chromoendoscopy with Lugol’s solution, a flat lesion of the esophagus with a diameter of 20 mm at approximately 28 cm from the incisor teeth was seen (Figure 1). The resection borders were marked with dual knife and after submucosal injection of indigo-carmine and sodium hyaluronate solution, and *en bloc *resection of the lesion 2-3 mm away from the margins was successfully performed. Intra- and post-procedural prophylactic coagulation with hemostatic forceps followed. Complications such as delayed bleeding or perforation did not occur after the ESD and the patient was discharged 2 days after the treatment. The specimen was pinned against a plate peripherally by stainless-steel pins and entirely immersed in formaldehyde overnight to preserve the tissue shape and configuration (Figure 2).

**Figure 3:**
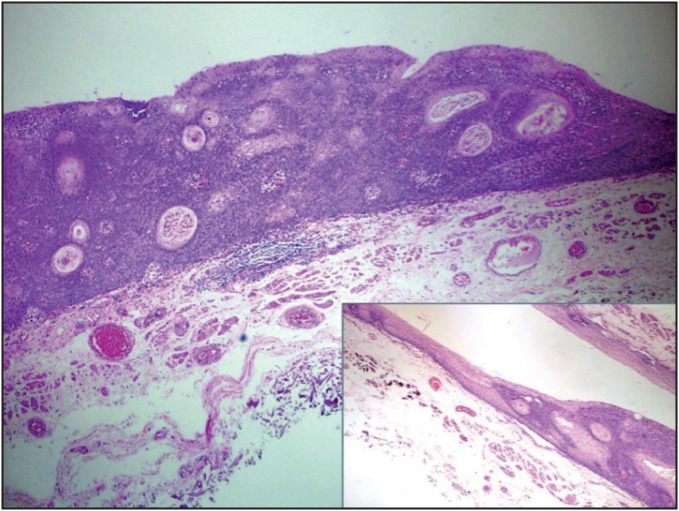
Circumscribed plaque-like lesion with acanthosis and the transition zone between the lesion and normal esophageal mucosa (inset) (H&E; x10).

**Figure 4:**
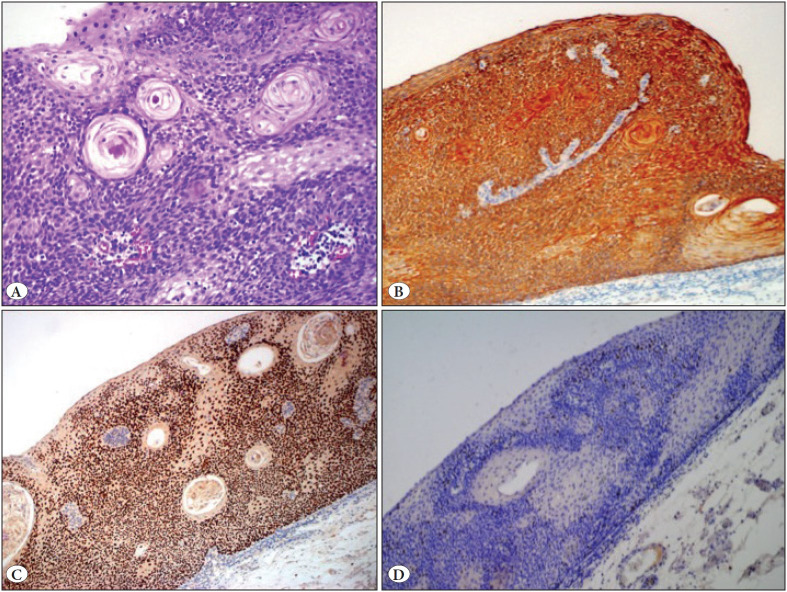
**A)** Squamous eddies, central keratinization and basaloid cells (H&E; x40). **B)** Cytokeratin 5/6 positivity (IHC; x20). **C)** Diffuse p63 positivity (IHC; x20). **D)** Ki-67 confined to basal layer (IHC; x20).

Macroscopic examination revealed a well circumscribed, slightly pigmented and elevated plague-like lesion with a diameter of 20 mm on a velvety mucosal surface. Alcian blue staining was used for macroscopic delineation of mucosal margins and the specimen was then serially sectioned perpendicularly at 2 mm intervals. All sections were subjected to histopathologic review. The lesion was well circumscribed, with plaque-like elevation on low power magnification and the base of the lesion was rough on an imaginary axis drawn between two mucosal-submucosal junctions at both ends of normal esophageal tissue. On 10x and 20x magnification, hematoxylin and eosin-stained sections revealed hyperkeratosis, acanthosis, and papillomatosis. Broad coalescing solid sheets and interconnecting trabeculae of basaloid cells were the consistent feature throughout the lesion (Figure 3). Squamous eddies and occasional central keratinization were present (Figure 4). Mitotic activity and koilocytes were not identified. Immunohistochemically, the lesion showed negativity with p16, diffuse positivity with cytokeratin 5/6, and diffuse nuclear positivity with p63 (Figure 4). The Ki-67 labeling index was confined only to the basal cell layer of the lesion and normal esophageal squamous epithelium (Figure 4). No dysplasia was identified.

## DISCUSSION

Seborrheic keratosis is one of the most common lesions seen by dermatologists and it is considered as a benign epidermal tumor but it may be a sign of concomitant skin cancer and internal malignancies. They are sharply demarcated, slightly elevated, hyperpigmented patch or plaque-like lesions and are seen commonly in areas such as the trunk, neck, face and upper extremities. They are considered as hyperkeratotic lesions of the epidermis and reported not to be seen on the mucosal surfaces (1). However, there are several case reports in the literature presenting seborrheic keratosis on the conjunctiva (4) and the nasal vestibule (5). All of the dermatological lesions with hyperkeratosis, acanthosis and papillomatosis might be considered in the clinical and pathological differential diagnosis. Our patient did not show any similar lesions on the skin and there was no internal malignancies detected with the imaging procedures performed before the endoscopic procedure. The Leser-Trélat syndrome, which is characterized by the eruptive appearance of multiple seborrheic keratoses in association with an underlying malignant disease, was therefore not considered during the differential diagnosis.

The term “seborrheic keratosis-like lesion” as a new entity was previously used in a case series by Talia and McCluggage that included a total of 7 cases of the cervix and vagina and a relationship with the human papilloma virus (HPV) was shown in two of these cases (6). However we did not find any similar lesions of the esophagus reported in the literature.

ESD is an effective method for neoplastic lesions of the esophagus and it is a safe treatment modality in the management of early esophageal squamous cell neoplasms. However, great skill in this technique is definitely required (2). ESD is suggested to be performed rather than EMR while dissecting lesions that are larger than 15 mm because it ensures *en bloc* resections and the recurrence rates are lower (7). A study by Chen et al. on 296 patients with early esophageal squamous cell neoplasms and high-grade intraepithelial neoplasms showed no cancer-related deaths and it was concluded that ESD is a well-accomplished and secure procedure (8).

Rare lesions of the esophagus resected with ESD or EMR reported in the literature include Barrett’s esophageal cancer (3), granular cell tumor (9) and leiomyomas in the category of stromal tumors (10).

Herein, we report a case of a superficial esophageal lesion resected with ESD. We diagnosed this morphologically benign case as a “seborrheic keratosis-like lesion of the esophagus” with the help of histopathologic and immunohistochemical findings. To our knowledge, this is the first case of the aforementioned lesion in the esophagus.

In conclusion, it should be kept in mind that seborrheic keratosis-like lesions might be rarely seen on mucosal surfaces such as the esophagus and that ESD is a safe procedure in *en bloc* resection of superficial esophageal lesions.

## Conflict of Interest

The authors declare no conflicts of interest.
